# Preparation and drug delivery of dextran-drug complex

**DOI:** 10.1080/10717544.2019.1580322

**Published:** 2019-03-11

**Authors:** Shiyu Huang, Gangliang Huang

**Affiliations:** Active Carbohydrate Research Institute, Chongqing Key Laboratory of Inorganic Functional Materials, Chongqing Normal University, Chongqing, China

**Keywords:** Dextran-drug complex, preparation, magnetic targeting, drug delivery

## Abstract

Dextran as a drug carrier for inhibiting cancer cells effectively reduces the toxic and side effects of the drug in the biological body. Targeting improves the concentration of active substance around the target tissue, which reduces damage to other heavy organs and other normal tissues. Dextran will be a potential carrier for the delivery of antitumor drugs in the future, which provides the possibility of slow-release chemotherapy and targeted drug delivery. Herein, the preparation and drug delivery of dextran-drug complex were summarized and discussed in detail.

## 1. Introduction

The biggest obstacle to the efficacy of drug is the side reaction. If the drug carrier is controlled, it can effectively enter the treatment site, avoid side reaction, reduce the damage of the original drug to important organs and normal cell tissues of the organism, and can fully play its due role (Sun, 2009). Herein, a series of examples of dextran as a drug carrier will be listed to illustrate the role of dextran in drug delivery.

Magnetically targeted drug delivery system with slow release and magnetic targeting is under the protection of dextran modification. The system has a high selectivity and they will be enriched near the target tissue or target cell (Huang & Huang, 2018). This reduces the damage of original drug to other normal tissue and normal cell, improves the therapeutic effect of the drug, and enhances the inhibition of tumor cells (Hashida et al., 1983).

Dextran-based gel-conjugated drugs have no toxic side effects and have a good material basis for the preparation of drug delivery systems (Takakura et al., 1984). As a drug-loading system, the dextran-based gel has the characteristics of large drug loading, convenient absorption, convenient use, and stable performance. These drugs can achieve long circulation in the blood circulation system and have certain targeting to tumor tissues. Release effect can reduce the toxic side effects of the original drug on the organism because of the biological characteristics of the dextran-based gel. It has become a hot spot in the field of drug delivery for controlled release and tissue engineering.

## 2. Preparation

In the design of new biomedical materials for triggered gene and drug delivery, modification of dextran-based polysaccharides is crucial (Hu et al., 2017). Shell-sheddable micelles based on dextran diblock copolymers could be used for efficient release of hydrophobic chemotherapeutics in cancer cells (Sun et al., 2010). Functionalized nonionic dextran scaffolds were prepared by atom transfer radical polymerization, which could be used to transfer genes effectively (Wang et al., 2011). The biodegradable comb-like gene vector from dextran skeleton had the biologically induced initiation site of atom transfer radical polymerization (Wang et al., 2012). The functionalized biodegradable dextran backbones were prepared using living radical polymerization, and the novel multifunctional nanoparticles obtained could be used to deliver therapeutic molecules (Duong et al., 2012). For an ideal polymer gene carrier, its serum stability is very important. Polycation carriers usually produce colloidal aggregation, which makes them easy to remove from bloodstream. It proved that incorporating zwitterionic betaine into multifunctional gene carriers was an effective method to produce serum-tolerant transfection carriers (Xiu et al., 2013). The galvanic replacement reaction is a useful method to prepare various hollow nanostructures. A simple synthesis of dextran-coated hollow Au-Ag nanoshell and its application in chemical thermotherapy were reported (Jang et al., 2014).

## 3. Lymphocytokinin-dextran conjugate (MMC-D)

Lymphatic metastasis is the most common form of metastasis of tumors, and its metastasis severely affects the treatment of cancer (Han, 1991). The most recent studies on chemotherapy drugs for lymphatic metastasis are polymer-targeted anti-tumor drugs. The focus of the research is to find drug-loading systems with greater selectivity and better therapeutic effects. In the study of this class of drugs, glucan and chitosan are used as carriers (Zhang, 1996).

Such conjugates are taken up by the lymphocyte’s specific uptake of a conjugate of a certain molecular size, which in turn aggregates the conjugate in the target tissue region (Li, 2014). Because this kind of conjugate has a large molecular volume and cannot enter the blood through the capillary wall, it can only be taken up by the macrophage or reticular epithelial cells through the pinocytosis, and the conjugate is taken into the whole body. Then, under the action of the enzyme, the pharmaceutically active substance is hydrolyzed, thereby killing the cancer cells (Huang, 2013). Therefore, such polymeric carrier-targeted drugs are most suitable for the treatment of lymph node tumors or lymphatic metastasis tumors, and the carrier itself is not antigenic.

**Figure 1.**
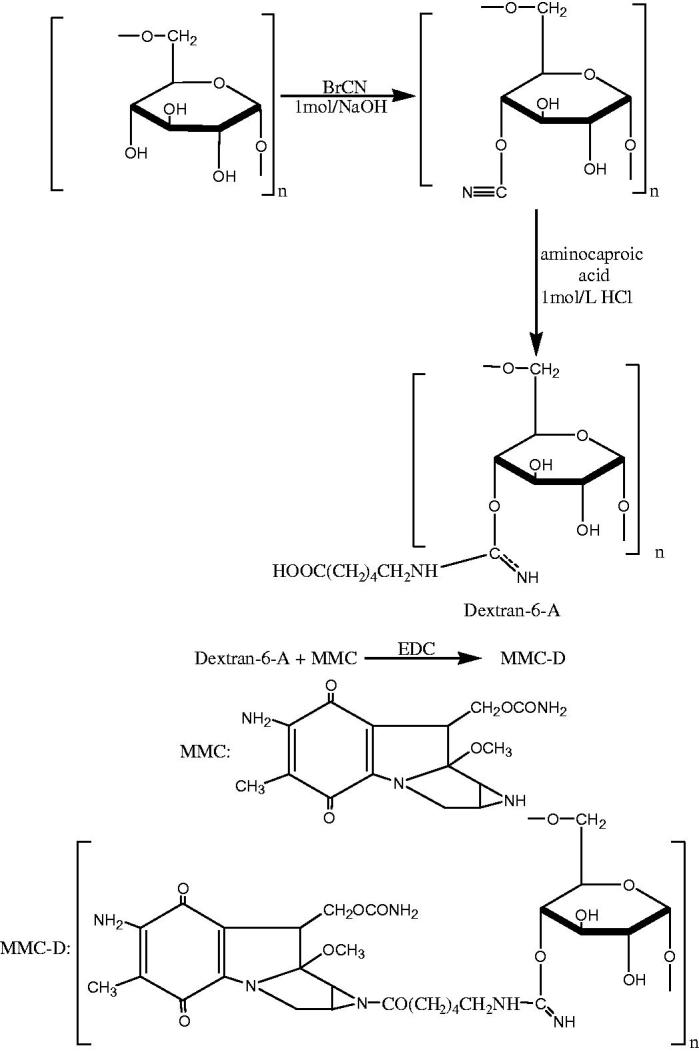
The synthetic route of MMC-D.

Mitomycin is a commonly used anti-tumor drug in the clinic. It has a strong antibacterial effect on various gram-positive bacteria, but its molecular weight is small, it will enter the blood through the capillary wall, and its toxic side effects are large. This cannot selectively play a role in lymph node metastasis. The local injection of dextran (T10-500) can selectively be taken up by macrophages or reticular epithelial cells through pinocytosis, and cannot enter the blood through the capillary wall, and can be quickly metabolized in the body. With a nontoxic and non-retention property, a certain molecular weight dextran and an anti-tumor small molecule drug mitomycin C (MMC) were coupled through a spacer to form a dextran-targeted antitumor drug (MMC-D) (Bao, 2010) (Figure 1).

The action process of MMC-D is as follows (Figure 2).

Figure 2.The action process of MMC-D.

**Figure:**
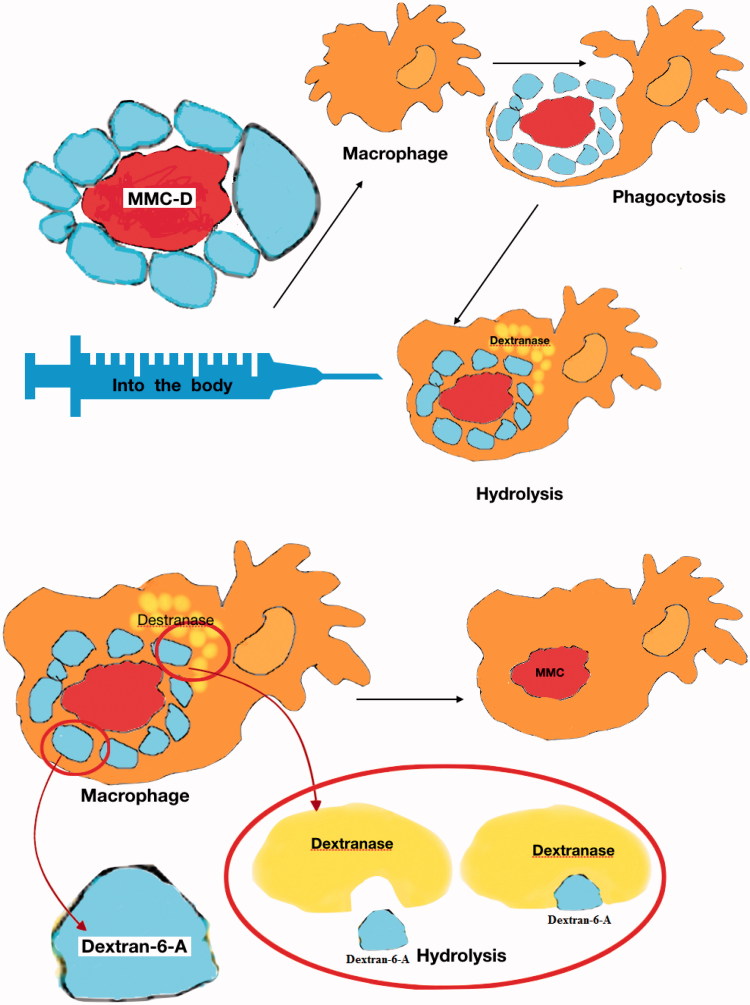

**Figure:**
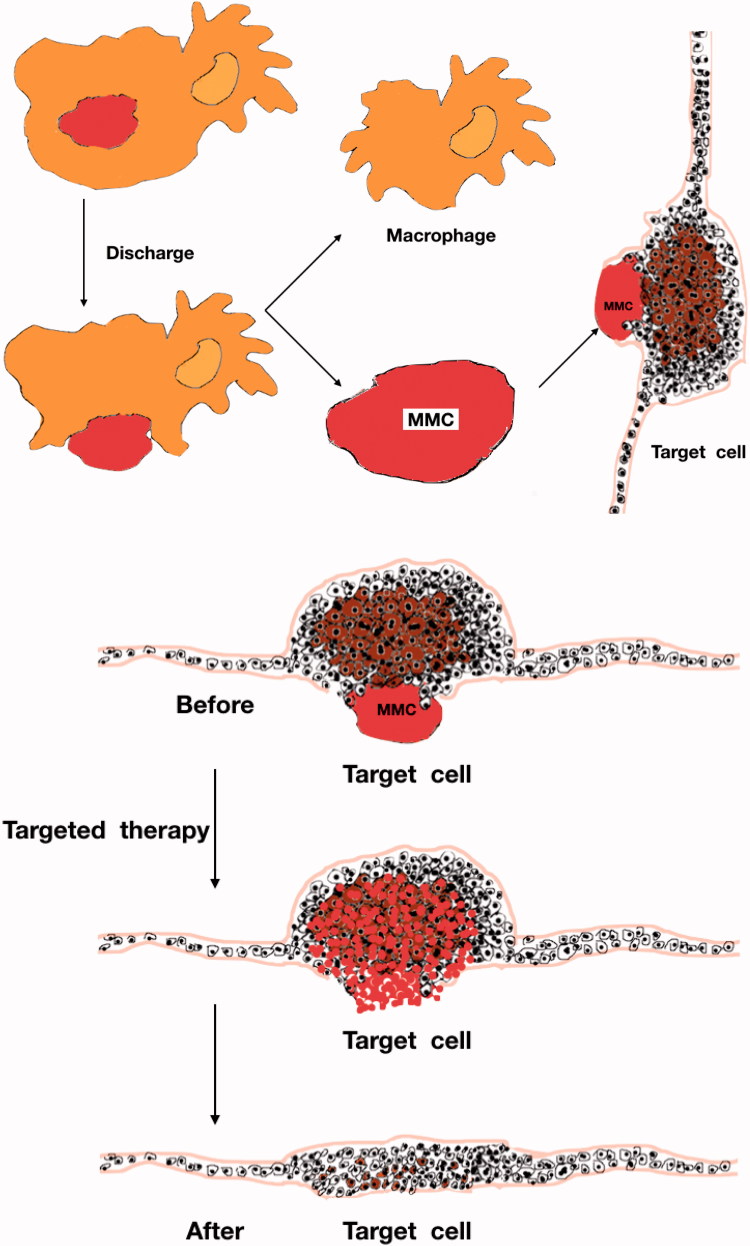

Because of its large size, MMC-D did not enter the blood through the capillary wall like MMC, but was ingested by macrophages or by pinocytosis. Then the dextran layer was hydrolyzed and the drug remained. The active substance MMC was excreted by macrophages, transported to cancerous tissues, and combined with cancer cells to kill them, or inhibited the continued division of cancer cells, so that target cells could be effectively treated (Yan & Zhong, 1999).

The results of MTT assay showed that MMC and MMC-D had certain killing effects on squamous cell carcinoma cells. The efficacy of MMC-D was slightly lower than that of MMC, and the effect was slower than MMC. It might be that MMC-D had a hydrolysis process *in vivo*, but it had a lasting effect.

It showed that both MMC and MMC-D had a strong apoptosis-inducing effect on cancer cell K562, had a considerable influence on the growth cycle of cancer cells, which was concentration-dependent. The biological activity of MMC-D was slightly lower than that of MMC, but it still retained strong activity. This result was consistent with the result of the MTT assay for the susceptibility test of MMC-D.

Followed by study of the biological properties of the conjugate, the conjugate was used for experimental oral cancer lymph node metastasis, the results demonstrated that MMC-D had a strong anti-tumor and lymph trend had obvious targeting.

## 4. A drug delivery system with controlled release and magnetic targeting under the protection of dextran modification

These magnetic targeted drug delivery systems have high selectivity, they will be enriched in the vicinity of the target tissues or target cells, reducing the damage of the original drug to other normal tissues and normal cells, improving the therapeutic effect of the drug, and enhancing the inhibitory effect on tumor cells.

### 4.1. Dextran-magnetic layered composite hydroxide-fluorouracil targeting lipid

Fluorouracil (FU) is a commonly used broad-spectrum chemotherapy drug, but the original drug has strong gastrointestinal reaction and short half-life (Gou et al., 2009a, 2009b). When FU was inserted into the layer of magnetic layered composite hydroxide (MLDH), a new type of dextran-MLDH-FU (DMF) with slow release and magnetic targeting was formed under the protection of dextran (Gou et al., 2009a, 2009b). The gastric cancer solid tumor model was established by subcutaneous injection of MGC-803 gastric cancer cells, and the cancer cells were allowed to proliferate normally in nude mice for 10 d. The tumor-bearing mice were randomly divided into four groups, and then intraperitoneally injected with normal saline, FU original drug, and DMFL, respectively. Intervention treatment was performed under both magnetic and non-magnetic conditions, and the diet and body weight of nude mice were observed and recorded.

Preliminary study on the inhibitory effect of DMFL on the proliferation of MGC-803 gastric cancer in nude mice, the results showed that the establishment of MGC-803 gastric cancer solid tumor model was short, had high success rate and rapid proliferation. DMFL had good magnetic targeting transport and slowing the effect of release chemotherapy, which could guide 5-FU to move *in vivo* through the magnetic field effect *in vitro*, and effectively enrich the tumor site, maintain a continuous and effective drug concentration around the target tissue, and enhance the inhibition and killing effect on tumor cells. The distribution of drugs in other organs was reduced, so as to achieve high-efficiency and low-toxic therapeutic effect. Compared with other groups, the mice in the DMFL magnetic field treatment group had good living conditions, no significant decrease in body weight, relatively slow tumor growth, and tumor weight relative to other groups. There were also significant differences, and DMFL chemotherapy did not cause significant damage to other important organs (Ermer et al., 2005).

To further investigate the mechanism of DMFL system inhibiting tumor proliferation, the expression of VEGF and Ki-67 protein in tumor tissues and the number of neovascularization in tumor tissues were determined by immunohistochemistry and immunofluorescence techniques, respectively.

Immunohistochemical analysis showed that the expression of neovascular and VEGF protein was significantly decreased after magnetic targeting therapy. The immunofluorescence results showed that the expression of Ki-67 protein was also significantly decreased in the magnetic therapy group. The results of the above studies indicated that carrying 5-FU in DMFL liposomes was more effective in blocking the conversion of deoxyuridine nucleotides to deoxythymidine nucleotides, and inhibiting VEGF expression or reducing VEGF at the same time. The active and weaken promotion of angiogenesis by this protein factor reduced the formation of new blood vessels, thereby blocking the nutrient source and substance exchange of cancer cells, enhancing the drug effect of 5-FU-induced tumor cell apoptosis and inhibiting cancer cell proliferation. The cancer cells were directly killed while increasing the chance of tumor tissue necrosis due to ischemia (Shi et al., 2007).

### 4.2. ‘The dextran-magnetic LDH-Fu’ transport model

The supramolecular assembly of ‘dextran-magnetic LDH-fluorouracil’ was used as a magnetic targeting drug delivery system (Zhao et al., 1998). To investigate the magnetic targeting of drug transport distribution in various tissues of rat models, a method for establishing an internal standard of high performance liquid chromatography was used to determine the concentration of drug distribution in various tissues of rats after administration. After intraperitoneal injection, the magnetic field was disturbed at different sites. The experimental results showed that the concentration of the drug in the rat where the magnetic field was established was significantly higher than that of the tissue away from the magnetic field (Gou et al., 2013). This result validated the *in vivo* magnetic field targeting of the three-stage supramolecular drug transport model. The magnetically targeted LDH-fluorouracil-dextran tertiary complex could accumulate in specific parts of the organism under the action of an external magnetic field, showing good magnetic targeting specificity.

The HPLC internal standard method for the determination of 5-Fu concentration in rat plasma after drug administration was established. The peak time of the LDH group was at 6 h, and the peak time of the original drug group was at 10 min. The LDH group was 36 times longer than the original drug group. The LDH group had a first half-life of 3.53 h, while the original drug group 5-Fu had an elimination half-life of 1.16 h, and the LDH group was 3.06 times that of the original drug group 5-Fu. The pharmacokinetic behavior of 5-Fu under the ‘dextran-magnetic LDH-fluorouracil’ transport model showed a good effect of sustained release *in vivo* (Deng et al., 2012).

Therefore, the ‘dextran-magnetic LDH-Fu’ transport model had good magnetic targeting specificity and good *in vivo* slow-release effect.

### 4.3. Supramolecular assembly of DMF delivery system

5-Fluorouracil (5-Fu) is clinically used for chemotherapy of malignant tumors (You, 2003). It is involved in the proliferative phase with the uptake of synthetic raw materials by cells, inhibiting cancer cell proliferation, altering cell dynamics and apoptosis-related gene expression during mitosis, and inducing apoptosis of tumor cells (Yan & Zhong, 1999). Oral 5-Fu drug has low bioavailability, poor absorption, and intense gastrointestinal reactions, and has a large side effect of injection. Through the ‘dextran-magnetic layered complex hydroxide-fluorouracil’ (DMF) system, administration could significantly reduce the side effects of 5-Fu and achieve targeted therapy.

In the DMF mode of administration, 5-Fu was slowly released by LDH laminate control, the amount of drug distributed to tissues and plasma would be reduced, and some conventional methods would be difficult to detect. HPLC is the primary method for separation and content determination of biological samples (WF, 2006). However, 5-Fu is also soluble in acid with strong polarity, poor retention column and short residence time. The separation of endogenous substances in biological samples is poor and the measurement conditions are difficult to grasp (Rollas et al., 2002; Bedia et al., 2006).

The dextran magnetic layered hydroxide-fluorouracil mixture was synthesized by co-precipitation intercalation *in situ* composite solvent conversion technology. It was characterized by X-ray powder diffraction, infrared spectroscopy, transmission electron microscopy, and thermogravimetric analysis. *In vitro* release experiments were carried out to study the phase characteristics and sustained release properties of DMF, and then the *in vivo* targeted and sustained release effects of DMF were investigated by animal experiments (Sun, 1999). The results show that the XRD of DMF was consistent with the diffraction characteristics of R-hexagonal layered composite hydroxide and Fd-3m cubic ferrite. Infrared spectra showed that DMF was a supramolecular complex composed of dextran, magnetic layered hydroxide, and fluorouracil. MLDH-FU had hexagonal and layered features. DEF could protect the layered structure of MLDH-FU, improve particle dispersion performance, and enhance the sustained release performance of the carrier system. The pharmacokinetics of DMF showed peak attenuation and multi-peak phenomenon of cycle growth. The highest peak of DMF was 1/37 of the original drug, and the bioavailability was 419% of the original drug (Brondsted et al., 1998).

‘The dextran-magnetic layered composite hydroxide-fluorouracil administration system supramolecular’ had certain stability and targeting specificity *in vivo*, and could continue to participate in the systemic circulation and magnetic targeting tissue enrichment in order to exert anti-tumor drugs. The release and release of Fu from DMF changed the existence of the original drug and triggered changes in pharmacokinetic parameters and bioavailability, which was a transport anti-tumor drug. Targeted and sustained release chemotherapy offered the possibility (Cheung et al., 2005).

## 5. Dextran-based gel-coupled drugs

Dextran-based gels have been widely used in the biomedical field due to their good mechanical strength and swelling degradation properties (Stenekes et al., 2001). Their preparation methods have attracted much attention (Basan et al., 2007).

At present, there are many preparation methods for gels and microgels, including crystallization, physical cross-linking chemical cross-linking, radiation cross-linking copolymerization, etc. The preparation of dextran hydrogels with the same properties is simple and stable as protein drug carriers (Bonneaux & Dellacherie, 1995). Physical cross-linking is another important method for the preparation of dextran-based hydrogels. There is no significant difference between hydrolyzed gels prepared by chemical cross-linking methods, but there are many advantages as drug carriers.

**Figure 3.**
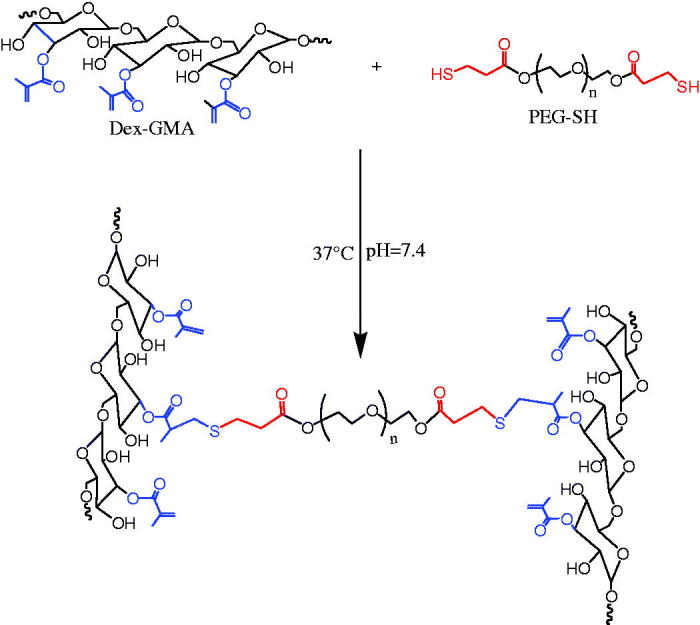
Preparation of Dex-GMA/PEG-SH(D/P) hydrogel.

Preparation of Dex-GMA/PEG-SH(D/P) hydrogel is as follows (Figure 3).

The method is simple and physically cross-linked by hydrogel. Glue has the following advantages: (1) it can avoid drug inactivation caused by chemical cross-linking; (2) this hydrogel can be synthesized at room temperature, in body fluids, acidity, alkalinity, and any liquid environment; and (3) it can be solidified *in vivo* after liquid injection. There is no special pH requirement during the degradation process (Muangsiri & Kirsch, 2006). It is reported the effect of various synthetic parameters of dextran-based hydrogel microspheres on the sphericity, and pointed out that different properties of dextran-based hydrogels can be prepared by changing the synthesis parameters. The ball is applied to different drug delivery systems. A dextran-based intelligent hydrogel with pH sensitivity can be obtained by dextran activation, chemical modification or endowing with corresponding functional groups, but the process is immature (Zhou et al., 2009). In future research, in addition to proper cross-linking of dextran or complexing with other natural or synthetic polymers, attention should also be paid to the chemical modification of dextran-based smart hydrogels. The modification and imparting new properties have led to the development of a series of dextran-based smart hydrogels, which have become the new carrier materials in the field of tissue engineering and controlled release for drug delivery (Gumargalieva et al., 1996).

Mehvar’s study on dextran gel proved that dextran gel was very stable in the stomach and small intestine, did not degrade, but could be degraded in the large intestine. After encapsulation of hydrocortisone, the drug release test within the first 3 h, there was 10% drug release, more than 3% of drug release within 24 h. The glucanase could make it completely and completely degraded, the drug was completely released. Dextran and diisocyanate cross-linked compound was formed into a hydrogel (Torchilin, 2000). *In vitro* and *in vivo* drug release experiments showed that the gel could be degraded by glucanase specific to the colon site, and it was found that the chemical composition of the gel could control the swelling property and mechanical strength of the gel in the digestive juice, ensuring that the drug was in the stomach (Muangsiri & Kirsch, 2006). The small intestine did not release or release a small amount of gel into the 6.6 mm hydrocortisone tablets, which was used for *in vitro* release experiments. The results showed that the release rate of the drug was mainly affected by the degree of cross-linking of the gel and glucanase (Tomlinson & Davis, 1986). The effect was related to the hydrophilicity of the drug itself. This diplomatic dextran could also be used as a capsule material.

The free radical co-polymerization technique was used to fabricate pH-sensitive nanogels (NG1) and TPGS-grafted nanogels (NG2), which were built with orthoester crosslinker (OEAM).

**Figure 4.**
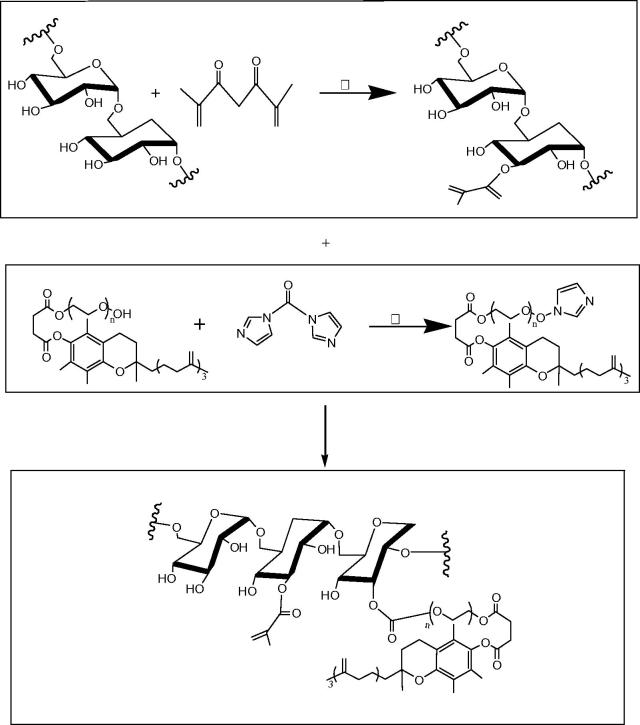
Synthesis of MA-DEX and TPGS-MA-DEX.

Synthesis of MA-DEX and TPGS-MA-DEX is as follows (Figure 4).

This capsule has been experimentally proven to have a good colon-specific release function. The dextran gel is used as a system for intestinal delivery. The drug delivery system is currently the most widely used (Schlemmer et al., 1999). It has no effect on long-term proliferation, and has good biocompatibility. Its protein encapsulation efficiency can reach 88%∼98%, and drug release can reach 30 d, which has obvious advantages over traditional carriers. It indicates its great application potential in the field of tissue engineering. The gel microspheres loaded with biomaterials such as chitosan gelatin have been successfully loaded with active factors and have shown good biological properties, but dextran-based hydrogels have not yet been applied. The field of load-active growth factor application and tissue engineering is still in the stage of envisioning and laboratory research (Farokhzad et al., 2004).

There are still many problems to be solved. There is no ideal preparation process at present, which can ensure satisfactory drug loading and encapsulation rate, and can greatly reduce the loss of biological activity of active factors during preparation (Koten et al., 2003). Foreign scholars have gained valuable experience in the controlled release of active factors, indicating that the use of controlled release of active factors to promote tissue regeneration has a broad research space with dextran-based hydrogel as a smart organism (Bos et al., 2004). Some characteristics are not realized by traditional carriers, it is believed that with the further study of dextran-based smart materials, controlled intelligent drug delivery in the field of tissue engineering is bound to be achieved.

Dextran gel has no toxic side effects, has a good material basis for the preparation of drug delivery systems, and acts as a drug delivery system (Cadee et al., 2001). The dextran-based gel has the characteristics of large drug loading, easy absorption, convenient administration, and stable performance. These drugs can achieve long circulation in the blood circulation system, and have a certain targeted release effect on tumor tissue. Because of the biological characteristics of dextran gel, it can weaken the toxic side effects of drugs and make it a hot topic in the field of drug controlled release and tissue engineering (Stenekes et al., 2000).

### 5.1. pH-sensitive dextran microgel coupled with hydroxycamptothecin

A novel dextran microgel coupled with hydroxycamptothecin (HCPT) with a hydrazide structure as a linkage was prepared, and the nanogel was coupled with HCPT (Berry et al., 2003). As a result, the drug-loaded gel formed a hydrophilic and extracellular hydrophilic micelle structure, which could achieve passive targeting of tumor tissues. The release rate of 5.63% drugs in pH 5.4 buffer was higher than that in pH 4.5 and pH 7.4 buffer. The inhibition rate of nanogel-conjugated drugs in mice in the same time was not obvious compared with HCPT, but the survival rate was significantly improved. The results showed that the nanogel conjugate was pH sensitive and could be used as a passive targeted release drug for tumor tissue, which showed reduced toxicity in mice and showed significant sustained release characteristics (Hsiue et al., 2002).

The hydroformylated PEG-grafted dextran and its coupled HCPT could be assembled into regular nanoparticles in the aqueous phase, and also had good water dispersion stability properties. It was obviously superior to other environments in the drug-like tumor environment, which could achieve long circulation in the blood circulation system, and had a certain targeted release effect on tumor tissues. It was hopeful to overcome the bladder toxicity of HCPT in clinical applications. The inhibition rate of tumors was not significantly improved compared with the original drug, but the toxicity of the conjugate was significantly decreased at high dose (Chen et al., 2005).

### 5.2. Dextran gel microspheres coupled to isoniazid

Since its use in clinical practice in 1952, isoniazid has been one of the first choices for the treatment of various tuberculosis (Holland et al., 2003), especially for the treatment of tuberculosis, and its water solubility is very good (Holland et al., 2004). However, isoniazid has adverse reactions such as neurotoxicity and hepatotoxicity, and its half-life is short (Seung et al., 2010). For this reason, in the past few decades, small molecule drugs such as the condensate of isoniazid and vanillin, Ftivazide and Glyconiazzid of glucoacetal have been developed (Soo et al., 2010; Jae et al., 2011). So far, the design, synthesis and screening research of such small molecule drugs has continued, but they are not targeted.

Studies have shown that microspheres with a particle size of about 7 μm can be targeted in the lungs (Kriven et al., 2004), and polymer carriers that are targeted drugs for the lungs are usually hydrophilic or water-soluble, receptive, nontoxic, and biodegradable (Xu et al., 2007).

Dextran is a biopolymer material. It is a glucose-derived polymer derived from sucrose. It has good water solubility, good biocompatibility, safety, and nontoxicity. It has been used as a plasma volume expander in clinical practice. Blood flow promoters and antithrombotics are also used as carriers for sustained release drugs or polymeric prodrug carriers (Coughlan et al., 2004; Huang et al., 2004).

Animal experiments have shown that both biodegradable and nondegradable dextran-based microgels can be safely brought into the body by subcutaneous injection (Chiu et al., 1999).

The aldehyde-based glycan gel obtained by oxidation of dextran gel (Sephadex) was reacted with isoniazid to form a hydrazide-bonded gel-based prodrug, which had a good sustained-release effect (Kim et al., 2000). The release rate in the buffer solution of 7.4 and in the blood was slower than in the buffer solution at pH 4.0. However, dextran gel is a white beaded granule with a millimeter scale and is only suitable for oral preparation.

### 5.3. Dextran-based hydrogel drug

Polysaccharide is the most abundant polymer in nature (Zhang et al., 2004). Polysaccharide hydrosol has no toxic side effects and has a good material basis for preparing drug release system. It can control the combination of drug and carrier by changing gel properties, such as swelling coefficient, diffusion property, etc. Combination methods include covalent coupling, electrostatic adsorption, and microcapsule encapsulation, etc. They can achieve controlled release (Park, 1999).

Chemically modified polysaccharide hydrogels have environmentally sensitive properties, and some physicochemical properties are mutated after being stimulated by external environmental changes such as temperature and pH. This is one of the current intelligent biomaterials in medical and biological materials. It has been widely used (Jeong et al., 2000).

Dextran is a water-soluble polysaccharide with abundant source, nontoxicity, and good biocompatibility. It is a macromolecular polymer composed of glucose linked by 1,6-α-d glucopyranose.

As a drug-loading system, dextran hydrogel has the characteristics of large drug loading, easy absorption, convenient administration, and stable performance, and is a hot spot in the field of controlled release for drug delivery and tissue engineering (Changez et al., 2004).

## 6. Conclusion

Dextran as a drug carrier plays an important role in targeted administration and controlled release. It reduces the toxic side effects of the original drug on the organism, and enriches the active drug around the targeted tissue. It also reduces damage to other important organs and other normal tissues, and greatly exerts the efficacy of anti-tumor drugs.
